# Size-controlled aerosol synthesis of silver nanoparticles for plasmonic materials

**DOI:** 10.1007/s11051-012-0870-0

**Published:** 2012-06-03

**Authors:** Juha Harra, Jouni Mäkitalo, Roope Siikanen, Matti Virkki, Goëry Genty, Takayoshi Kobayashi, Martti Kauranen, Jyrki M. Mäkelä

**Affiliations:** 1 Aerosol Physics Laboratory, Department of Physics, Tampere University of Technology, P.O. Box 692, 33101 Tampere, Finland; 2 Optics Laboratory, Department of Physics, Tampere University of Technology, P.O. Box 692, 33101 Tampere, Finland; 3 Advanced Ultrafast Laser Research Center and Department of Engineering Science, University of Electro-Communications, Chofugaoka 1-5-1, Chofu, Tokyo 182-8585 Japan; 4 Core Research for Evolutional Science and Technology (CREST), Japan Science and Technology Agency, K’s Gobancho 7, Gobancho, Chiyoda-ku, Tokyo, 102-0076 Japan

**Keywords:** Aerosol synthesis, Silver nanoparticle, Localized surface plasmon resonance

## Abstract

Aerosol techniques were used to synthesize spherical and monodisperse silver nanoparticles for plasmonic materials. The particles were generated with an evaporation–condensation technique followed by size selection and sintering with a differential mobility analyzer and a tube furnace, respectively. Finally, the nanoparticles were collected on a glass substrate with an electrostatic precipitator. The particle size distributions were measured with a scanning mobility particle sizer and verified with a transmission electron microscope. A spectrophotometer was used to measure the optical extinction spectra of the prepared samples, which contained particles with diameters of approximately 50, 90 and 130 nm. By controlling the particle size, the dipolar peak of the localized surface plasmon resonance was tuned between wavelengths of 398 and 448 nm. In addition, quadrupolar resonances were observed at shorter wavelengths as predicted by the simplified theoretical model used to characterize the measured spectra.

## Introduction

Nanoparticles are widely studied and utilized in many scientific fields due to their unique properties, which differ from the corresponding bulk material. Optical properties of noble metal nanoparticles, such as silver and gold, are dominated by coherent oscillations of conduction electrons which give rise to localized surface plasmon resonances (LSPR) (Kreibig and Vollmer 1995; Halas 2010). These resonances can be tuned to a desired wavelength by changing the particle size, shape and dielectric environment (Kelly et al. 2003). Such tunability is essential for several interesting applications of nanophotonics, including surface-enhanced Raman spectroscopy (Nie and Emory 1997), optical data storage (Ditlbacher et al. 2000), cancer imaging and therapy (Loo et al. 2005), biomarkers (Sotiriou et al. 2011), biosensors (Hoa et al. 2007), plasmonic solar cells (Catchpole and Polman 2008) and colored coatings (Beyene et al. 2011).

Various techniques have been used to prepare both silver and gold nanoparticles for optical studies, for example ion implantation (Stepanov 2011), sputtering (Xu et al. 2005), pulsed laser deposition (Donnelly et al. 2006) and wet chemical techniques (Liz-Marzán 2004). These techniques usually offer very poor and often rather restricted size control of spherical nanoparticles which results only in relatively small particles with diameters less than ∼30 nm. However, the size range of spherical nanoparticles generated in the gas phase with aerosol techniques covers practically the whole nanoscale from a few nanometers to over 100 nm. This gives the opportunity to tune the LSPR more extensively. In addition, aerosol synthesis requires neither expensive vacuum technology nor liquid solvents. Aerosol processes are also continuous, scalable, and the generated nanoparticles can be directly deposited on the desired substrate. Therefore, aerosol synthesis offers high potential for preparing nanoparticles for plasmonic materials.

Different aerosol synthesis techniques of silver nanoparticles have been widely studied (Mäkelä et al. 2004; Ku and Maynard 2006; Tabrizi et al. 2009). In addition, silver nanoparticle samples for different types of applications have been previously prepared with aerosol techniques. For example, Deppert et al. (1994) deposited spherical silver particles on a semiconductor to serve as etch masks for quantum-dot structures and Kang et al. (2004) prepared patterns consisting of silver nanoparticles for electronic devices. More recently, Sotiriou et al. (2010) used a flame aerosol method to prepare silica coated silver particles for plasmonic biosensors. The silica coating prevented flocculation of the silver particles when dispersed in aqueous suspensions. Thus, the plasmon spectrum was tuned by controlling the amount of silica in the samples. However, aerosol techniques, especially the particle size control aspects, have not been fully exploited in the preparation of plasmonic materials.

In the present study, aerosol techniques were used to prepare plasmonic materials. The prepared samples consisted of spherical and monodisperse silver nanoparticles with diameters of approximately 50, 90 and 130 nm deposited on a glass substrate. As a result of the particle size control, the dipolar resonance of the samples was tuned within the wavelengths 398–448 nm. Furthermore, the measured extinction spectra displayed additional peaks at shorter wavelengths. The theoretical calculations confirmed that these peaks are most likely quadrupolar resonances.

## Experimental

**Fig. 1:**
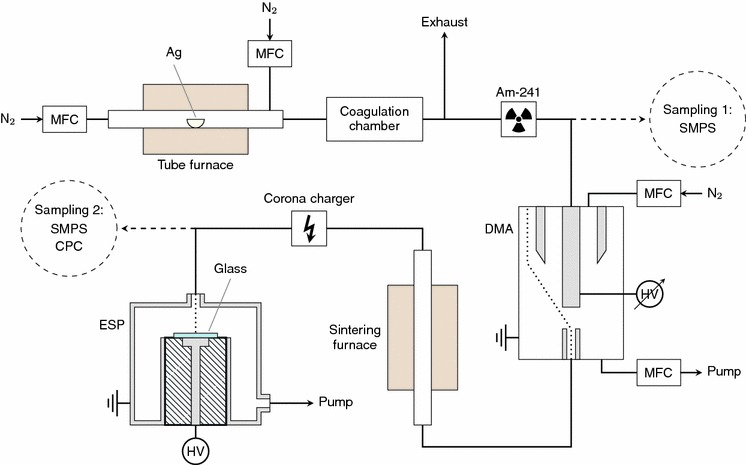
The experimental setup for preparing silver nanoparticle samples

Figure 1 shows a schematic diagram of the entire experimental setup to prepare the silver nanoparticle samples. The particles were generated using an evaporation–condensation technique introduced by Scheibel and Porstendörfer (1983). A ceramic crucible containing a small amount of bulk silver was placed inside the ceramic worktube of a tube furnace (Carbolite TZF 15/50/610). The silver was evaporated at the center of the furnace at temperatures between 1,300 and 1,400 °C. The formed metal vapor was first carried out of the furnace and then diluted with inert gas streams of nitrogen (N_2_). Both flow rates were set at 3 L/min with mass flow controllers (MFC). Because of the sudden temperature decrease after the furnace, the silver vapor condenses to form primary nanoparticles, which then coagulate to form agglomerates. According to previous studies with similar particle generation setup, the chain-like silver agglomerates consist of spherical primary particles with diameters of approximately 20 nm (Weber and Friedlander 1997; Ku and Maynard 2006). In the present study, coagulation was enhanced by placing a coagulation chamber with a volume of 8.5 L in the flow line.

After the chamber, the aerosol passed through a radioactive Am-241 source, which was used for bipolar charging of the particles. A narrow particle size distribution was then selected from the aerosol with a differential mobility analyzer (DMA) (Knutson and Whitby 1975). This standard instrument classifies particles with an electric field according to their electrical mobility, which is a function of the size and charge of an aerosol particle. The selected particles are mostly singly charged, either negatively or positively. The mean size and the width of the selected size distribution can be altered by changing the voltage and flow rates in the DMA. The sheath and excess flow rates of the Vienna type DMA used in this study were set to 10 L/min with MFCs, while the sample flow rate was 1.5 L/min. After the DMA, the size-selected agglomerates were sintered to spherical nanoparticles in an additional tube furnace (Schmidt-Ott 1988; Karlsson et al. 2005). A quartz worktube was used in the sintering furnace (Carbolite MTF 12/38/400), and the temperature of the furnace was set to 800 °C.

Even though the size-selected and sintered silver particles were already charged, their charge level was increased with a unipolar corona charger to ensure that the electrical mobility of the particles is high enough for electrical collection. An electrostatic precipitator (ESP), similar to the one studied by Krinke et al. (2002), was used to collect the silver nanoparticles on a substrate. The cylinder shaped ESP had an inlet with a diameter of approximately 5 mm which widened to 100 mm inside the ESP. This caused the velocity of the particles to decrease, while a nearly perpendicular electric field of 2.5 kV/cm pulled the charged particles on a substrate which was placed on a round electrode with a diameter of 20 mm. Due to the increased particle charge obtained in the corona charger, the collection efficiency of the ESP exceeded 90 %. The substrates were standard 1 mm thick microscope glass slides. In addition, particles were collected on carbon-coated copper TEM grids for electron microscopy.

A scanning mobility particle sizer (SMPS) (Wang and Flagan 1990) consisting of a DMA (TSI Model 3081) and a condensation particle counter (CPC, TSI Model 3025) was used to measure particle size distributions in the gas phase both before and after the size selection at sampling 1 and sampling 2, respectively. The CPC alone measured the number concentration of the size-selected and sintered particles at sampling 2. After the collection, the nanoparticles were studied with a transmission electron microscope (TEM, Jeol JEM-2010), and particle size distributions were determined from the TEM images with an image processing program ImageJ (Abràmoff et al. 2004). In addition, a scanning electron microscope (SEM, Zeiss ULTRA PLUS) was used to study the actual glass samples with the deposited particles. The extinction spectra of the samples were measured with a UV–VIS spectrophotometer (Shimadzu UV-3600). A clean microscope glass slide was used as a reference sample.

## Results and discussions

### Aerosol measurements

**Fig. 2:**
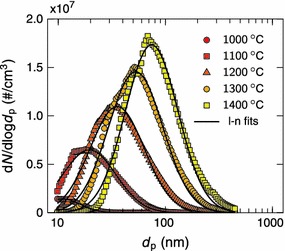
Size distributions of silver agglomerates for different furnace temperatures. A log-normal distribution has been fitted to each measurement

Figure 2 shows the original particle size distribution of silver agglomerates before the size selection for different furnace temperatures at sampling 1. The measured distributions were log-normally distributed ranging from a few nanometers to hundreds of nanometers depending on the temperature of the tube furnace. When the temperature was increased from 1,000 to 1,400 °C, the geometric mean diameter of the size distribution increased approximately from 10 to 70 nm. In addition, the total concentration of the particles increased by approximately two orders of magnitude from 10^5^ to 10^7^ #/cm^3^. Both increases, the particle size and concentration, resulted from the higher evaporation rate of silver at higher temperatures. In order to avoid extensive collection times during the sample preparation, it was preferable to have high particle concentration. Therefore, the tube furnace was set to temperatures of 1,300−1,400 °C during the sample preparation.

**Fig. 3:**
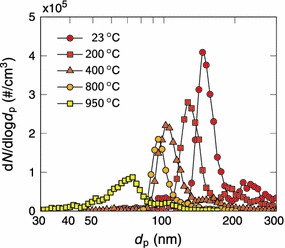
Size distributions of size-selected silver particles sintered at different temperatures. The temperature of the tube furnace was 1,350 °C

Size distributions of the size-selected and sintered particles were measured with the SMPS at sampling 2. Figure 3 shows typical examples of the particle size distributions for different sintering temperatures. When the sintering temperature was increased, the particle concentration decreased. In addition, the size distribution shifted to smaller particle sizes and became slightly narrower. However, at temperatures near the bulk melting point of silver (962 °C), the size distribution clearly broadened. It should also be noted that there is a second, much weaker, particle mode with a larger particle size. This mode partly merges into the main mode when the temperature is increased. These larger particles are most likely doubly charged by the radioactive source. Therefore, they have the same electrical mobility as the desired particles. This results in their selection in the DMA. In order to eliminate these particles and to narrow the size distribution before the collection, another DMA could be used after the sintering furnace for a second size selection. However, that might notably lower the particle concentration and lead to extended collection times.

**Fig. 4:**
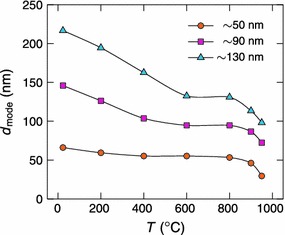
The mode size of the size-selected silver particles as a function of the sintering temperature. At 800 °C, the particles are spherical with diameters of approximately 50, 90 and 130 nm

Three particle sizes were chosen for the sample preparation. Agglomerate sizes of approximately 70, 150 and 220 nm were selected with the DMA and sintered to spherical nanoparticles with diameters of approximately 50, 90 and 130 nm, respectively. For the preparation of the 50, 90 and 130 nm particles, tube furnace temperatures of 1,300, 1,350 and 1,400 °C, respectively, were used. Figure 4 shows the mode sizes of the particle size distributions as a function of the sintering temperature for the three size-selected particle sizes. According to Karlsson et al. (2005), the sintering process can be divided into three steps: compaction, internal rearrangement and evaporation, which are also evident in Fig. 4. First, at near room temperatures, the diameter of the agglomerates begins to decrease due to compaction. After the sintering temperature exceeds approximately 300−500 °C, depending on the particle size, the particle diameter stays constant. This means that the compaction has finished. However, internal rearrangements still occur, which lead to spherical particles with higher degree of crystallinity. Finally, at temperatures near the bulk melting point of silver, the particle size suddenly decreases again because of evaporation as reported by Schmidt-Ott (1988). To ensure that the silver nanoparticles were as spherical as possible without excess losses, the sintering temperature was set to 800 °C for the sample preparation.

**Fig. 5:**
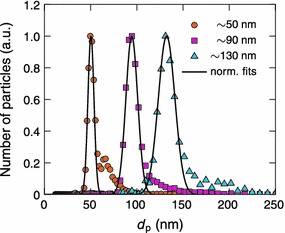
Normalized size distributions of the size-selected silver particles sintered at 800 °C. The total particle number concentration was $4\,\times\,10^4, 3\,\times\,10^4$ and $1.5 \times 10^{4}\, \#/\hbox{cm}^3$ for the 50, 90 and 130 nm particles, respectively. A normal distribution has been fitted to each measurement

Figure 5 shows the final normalized size distributions of the three size-selected and sintered particle sizes before the collection. Normal distributions were fitted to the size distributions with mean diameters of 50.3, 94.5 and 132.2 nm and relative standard deviations of 9.1, 9.5 and 9.6 %, respectively. The small tails caused by the doubly charged particles were not taken into account in the fitting.

### Electron microscopy

**Fig. 6:**
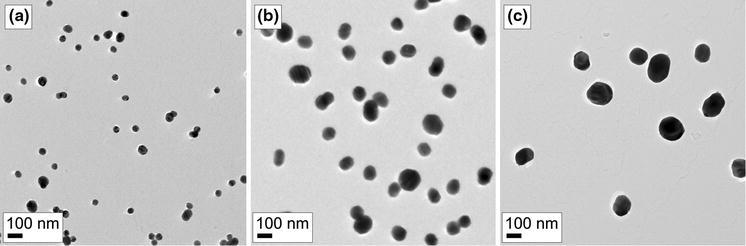
TEM images of size-selected and sintered silver nanoparticles with diameters of approximately **a** 50, **b** 90 and **c** 130 nm

Figure 6 shows TEM images of the size-selected and sintered silver nanoparticles collected on carbon-coated copper TEM grids. The sintering was successful, since most of the particles are spherical. However, deviations from spherical shape can be noted with some of the larger silver particles, which are slightly elongated. In addition, some of the particles are attached to each other, most likely during the collection process, and form agglomerates consisting of two or more spherical particles. This means that when the particle coverage on the substrate is increased, the fraction of separated particles will decrease. Moreover, if the spherical particles have a galvanic connection to each other, the optical properties of the nanoparticle samples most likely differ from an ideal situation. A possible way to prevent these galvanic connections would be to coat the silver particles with a dielectric material in the gas phase before the collection. This method could also be used to change the dielectric environment of the particles.

The collection times for the 50, 90 and 130 nm silver particles in the TEM samples were 163, 68 and 57 min, respectively. Rough estimates of the particle coverage on the substrate were made before the collection based on the particle concentration measured with the CPC, the flow rate in the ESP, and the size of the collection electrode. It was assumed that the particles are monodisperse and that the collection was ideal. The estimated particle coverage in all the TEM samples was 6 %, while the coverages calculated from several TEM images for the 50, 90 and 130 nm particles were 4.2, 7.4 and 3.4 %, respectively. The measured coverages are reasonably close to the rough estimate. The reason for the low coverage of the 130 nm particles can be explained with non-ideal collection due to the low electrical mobility of the larger nanoparticles. It was evident that the collection area of the 130 nm particles was slightly larger than the area of the collection electrode.

**Fig. 7:**
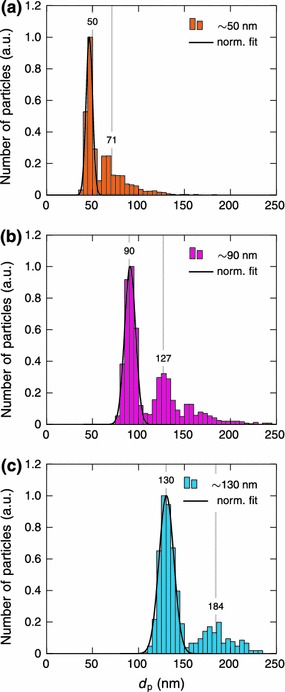
Size distributions of size-selected and sintered silver nanoparticles with diameters of approximately **a** 50, **b** 90 and **c** 130 nm. The distributions were calculated from several TEM images assuming that particles are spherical. The number of counted particles was 2546, 1289 and 594 for the 50, 90 and 130 nm particles, respectively

Particle area distributions in the TEM samples were determined from several TEM images and converted to particle size distributions assuming spherical shape. Figure 7 shows size distributions of the three different samples shown in Fig. 6. Clearly all of the size distributions have two distinctive modes. The largest peaks at particle sizes of 46.6, 90.9 and 130.4, with relative standard deviations of 10.7, 9.2 and 8.7, respectively, correspond to separated spherical particles. The particle sizes and deviations are in good agreement with the SMPS measurements in Fig. 5. The second mode is partly caused by the tail extending to larger particles observed in the SMPS measurements. However, the main reason is the agglomeration on the substrate during the collection process as discussed earlier. In Fig. 7, the vertical lines, near the peaks of the second modes, at particle sizes of 71, 127 and 184 nm correspond to two attached spherical 50, 90 and 130 nm particles, respectively.

**Fig. 8:**
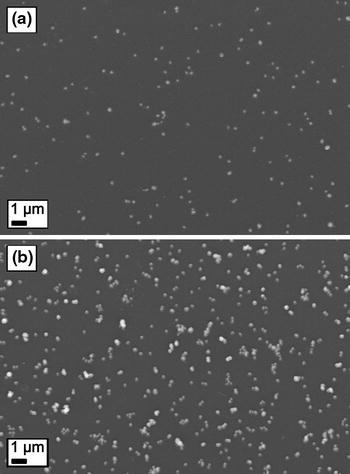
SEM images of 90 nm silver particles on a glass slide with a surface coverage of approximately **a** 2 and **b** 8 %

Figure 8 shows SEM images of the actual samples containing 90 nm silver particles on a glass slide with surface coverages of approximately 2 and 8 %. In the sample with 2 % coverage, the particles are mostly separated whereas the sample with 8 % coverage contains also agglomerates with sizes up to a few hundred nanometers. Sotiriou et al. (2010) found micrometer sized silver agglomerates from nanosilver films on a glass slide. These large agglomerates were formed within an aqueous suspension. However, no similar large silver agglomerates can be found in Fig. 8b and the particles are rather homogeneously dispersed, most likely because in this study the particles were deposited directly on the glass substrate from the aerosol phase. Direct deposition was also applied by Mädler et al. (2006) in the preparation of tin dioxide films using a flame aerosol method. These films were homogeneous and highly porous with a thickness of up to a few tens of micrometers. Evidently, the process yield in flame techniques is much higher than in the more delicate size selective furnace technique used in this study, in which the total process yield was estimated to be on the order of 10 μg/h.

### Comparing the optical measurements with the theoretical calculations

**Fig. 9:**
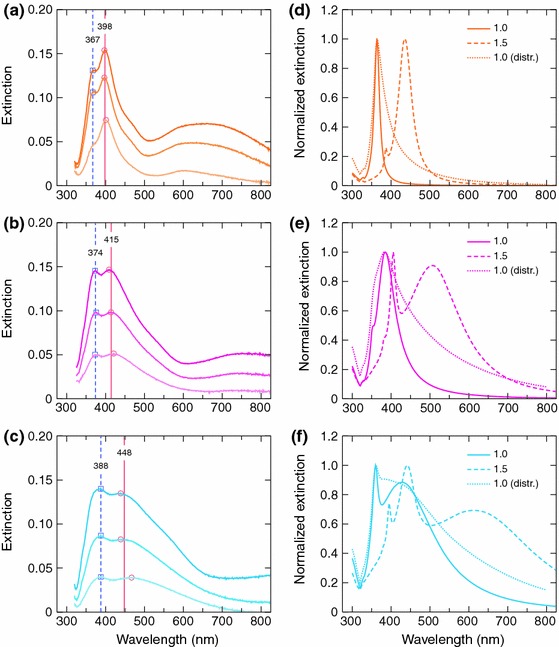
Measured extinction spectra of the silver nanoparticle samples with particle sizes of approximately **a** 50, **b** 90 and **c** 130 nm and calculated theoretical extinction spectra of spherical silver particles with diameters of **d** 50, **e** 90 and **f** 130 nm. The three measured spectra in **a**–**c** correspond to different particle coverages. Theoretical spectra in **d**–**f** were calculated for single particle using refractive indices of 1.0 and 1.5 and for particle size distribution using a refractive index of 1.0 for the dielectric environment

Figure 9a–c show extinction spectra of the prepared silver nanoparticle samples with particle diameters of approximately 50, 90 and 130 nm, respectively. Three extinction spectra are shown with each particle size. These spectra correspond to samples with different particle coverages ranging approximately from 2 to 8 %. Obviously, as the particle coverage on the substrate increases, the extinction increases as well.

For qualitative analysis of the measured extinction spectra, the systems were modeled by using a classical electrodynamical approach. A single homogeneous spherical particle in a homogeneous background medium was assumed and the excitation source was modeled as a time-harmonic plane wave. The solution for the problem was obtained by using Mie theory (Bohren and Huffman 1998), and finally the extinction cross-sections were deduced. Two refractive indices, 1.0 and 1.5, were used for the dielectric background medium to examine two extreme cases where the surrounding medium is air or the glass substrate. In addition to single particles, the cross-sections for distributions of spherical particles were calculated by using the size distributions from Fig. 7. The calculated spectra are shown in Fig. 9d–f, for silver particles with diameters of 50, 90 and 130 nm, respectively.

The measured extinction spectra display multiple peaks, of which two are near 400 nm and one at a longer wavelength. By comparing the measured spectra to the two single particle theoretical spectra, it can be concluded that the peaks (peak 1) at wavelengths of 398, 415 and 448 nm for the 50, 90 and 130 nm particles, respectively, are most likely dipolar LSPR peaks. The positions of these peaks fit between the dipolar resonance peaks of the two extreme cases. In addition to the red-shift of approximately 50 nm, the dipolar LSPR peaks broaden with increasing particle size, as expected from the theoretical calculations. However, the peaks in the measured spectra are not as sharp as implied by the theoretical calculations. This discrepancy can be explained with the low particle coverage in the samples. The average distance between the particles is roughly of the order of the wavelength and the samples contain particle free areas which lead to absorption flattening (Duysens 1956).

In addition to the dipolar resonances, the measured spectra show extinction peaks (peak 2) at shorter wavelengths of 367, 374 and 388 nm for the 50, 90 and 130 nm particles, respectively. Similar resonance peaks, which increase with particle size and refractive index, can be observed in the theoretical results. These peaks are quadrupolar plasmon resonances, as was verified by the theory. For the 50 nm particles, the theory is unable to explain the extinction peak in the measured spectrum as the quadrupolar resonance is very weak. For the larger particles, the quadrupolar resonance most likely contributes to the measurements.

**Fig. 10:**
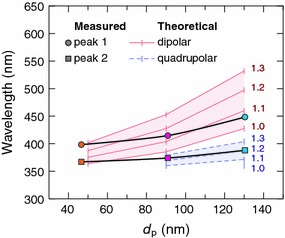
The wavelengths of the extinction peaks as a function of the particle diameter. The theoretical dipolar and quadrupolar LSPR peaks for different refractive indices of the background medium were calculated from the single particle model

In Fig. 10, the wavelengths of peaks 1 and 2 are presented as a function of the particle diameter together with the theoretical wavelengths of the dipolar and quadrupolar peaks for dielectric indices of 1.0–1.3. Even though the particle size dependence of the measured peaks differs from the theoretical resonances, these results strengthen the previous hypotheses, i.e. the dipolar and quadrupolar resonances clearly contribute to the measured peaks.

The broad extinction peaks in the measured spectra at longer wavelengths, approximately at 650, 750 and over 800 nm for 50, 90 and 130 nm particles, respectively, are not visible in any of the theoretical results. The extinction of these peaks as well as the ones below 400 nm becomes stronger in relation to the dipolar resonances with increasing particle coverage. This implies that the extinction peaks are likely related to the agglomeration of the particles during the collection.

The theoretical calculations that use the particle distributions instead of single particle show better correspondence to the measurements for the long wavelength part of the dipolar resonance peak. They also suggest that higher order resonance peaks are nearly indistinguishably merged to the dipolar resonance peaks. However, even these calculations cannot fully explain the extinction peak of the 50 nm particles below 400 nm and the peaks over 600 nm.

The theoretical model predicts the main characteristics of the prepared silver nanoparticle samples, i.e. the locations of the dipolar plasmon resonances. Discrepancies occur mostly for two reasons. First, to keep the model simple, the dielectric substrate has been neglected even though the substrate is known to significantly red-shift plasmon resonances (Malinsky et al. 2001). To some extent, this effect can be accounted for by tuning the refractive index of the surrounding medium but for quantitative analysis this is insufficient. Second, the real particle distributions also contain some non-spherical particles as shown in Fig. 6. Prolate ellipsoids and elongated bar shaped particles have been shown to exhibit plasmon oscillations along distinctive particle dimensions with significantly different resonance wavelengths (Gotschy et al. 1996; Kooij and Poelsema 2006). In addition, dimers of metal nanoparticles display different plasmon resonances for incident wave polarizations parallel and perpendicular to dimer axis. The wavelengths of these resonances are red and blue-shifted, respectively, in comparison to the single particle resonance (Atay et al. 2004).

## Conclusions

Plasmonic silver nanoparticle samples were prepared with aerosol techniques. The preparation technique consisted of a continuous chain of consecutive steps in the gas phase. First, agglomerated silver particles were generated in a tube furnace with an evaporation–condensation technique. The particle generation was followed by a selection of a narrow particle size distribution with a differential mobility analyzer and a sintering step in an additional furnace to obtain spherical particles. Finally, the size-selected and spherical silver nanoparticles were collected on glass substrates with an electrostatic precipitator.

The size of the silver particles calculated from the transmission electron microscope images was consistent with the measurements in the gas phase with the SMPS. This shows that the particle size control, which is important for tuning the localized surface plasmon resonance, can be very accurate when using aerosol techniques. In the present study, the size of the particles ranged within approximately 50–130 nm with a relative standard deviation close to 10 %. However, a small tail caused by doubly charged particles was also observed in the size distributions. At the expense of the collection time, it might be possible to further narrow the particle size distribution.

Since chain-like silver agglomerates were not present in the electron microscopy images and most of the particles were spherical, the sintering was effective. However, some of the larger nanoparticles, with diameters over ∼100 nm, were slightly elongated. The authors are not aware of any prior work that contains aerosol synthesis of spherical silver particles with diameters over 100 nm. Therefore, it is possible that the sintering step might set some limitations to the synthesis of large and completely spherical silver nanoparticles. The electron microscopy images also revealed that the particles attach to each other during the collection and form dimers, trimers, etc. on the substrate. These unwanted contacts could be eliminated by coating the nanoparticles in the gas phase with dielectric material before the collection.

The dipolar resonance of the prepared silver nanoparticle samples was tuned from 398 to 448 nm by changing the particle size in the samples. In addition to the dipolar resonance peak, other peaks were observed in the extinction spectra. The quadrupolar resonances evidently contributed to the extinction, especially with the larger particles. However, the dipolar and quadrupolar resonance peaks predicted by the simplified model were not able to explain all the characteristics of the measured extinction spectra, most likely because the theoretical model did not take into account particle dimers, elongated particles and the effects caused by the substrate.

All in all, plasmonic silver nanoparticle samples were successfully prepared. Furthermore, the possibility to control the size of the nanoparticles over a wide size range with the help of aerosol techniques improves the tunability of the localized surface plasmon resonance. Consequently, aerosol techniques offer high potential for tailoring nanoparticles for plasmonic materials.
